# Assessing effectiveness and implementation of a perioperative enhanced recovery protocol for children undergoing surgery: study protocol for a prospective, stepped-wedge, cluster, randomized, controlled clinical trial

**DOI:** 10.1186/s13063-020-04851-9

**Published:** 2020-11-16

**Authors:** Mehul V. Raval, Erin Wymore, Martha-Conley E. Ingram, Yao Tian, Julie K. Johnson, Jane L. Holl

**Affiliations:** 1 Surgical Outcomes Quality Improvement Center, Northwestern University Feinberg School of Medicine, 633 N. St. Clair, 20th Floor, Chicago, IL 60611 USA; 2 Division of Pediatric Surgery, Department of Surgery, Northwestern University Feinberg School of Medicine, Ann & Robert H. Lurie Children’s Hospital, 225 E. Chicago Ave, Chicago, IL 60611 USA; 3 Biological Science Division, University of Chicago, 5841 S. Maryland Avenue, Chicago, IL 60637 USA

**Keywords:** Pediatric enhanced recovery protocol, Gastrointestinal surgery, Implementation, Quality improvement

## Abstract

**Background:**

Perioperative enhanced recovery protocols (ERPs) have been found to decrease hospital length of stay, in-hospital costs, and complications among adult surgical populations but evidence for pediatric populations is lacking. The study is designed to evaluate the adoption, effectiveness, and generalizability of a 21-element ERP, adapted for pediatric surgery.

**Methods:**

The multicenter study is a stepped-wedge, cluster-randomized, pragmatic clinical trial that will evaluate the effectiveness of the *EN*hanced *R*ecovery *I*n *CH*ildren *U*ndergoing *S*urgery (ENRICH-US) intervention while also assessing site-specific adaptations, implementation fidelity, and sustainability. The target patient population is pediatric patients, between 10 and 18 years old, who undergo elective gastrointestinal surgery. Eighteen (*N* = 18) participating sites will be randomly assigned to one of three clusters with each cluster, in turn, being randomly assigned to an intervention start period (stepped-wedge). Each cluster will participate in a Learning Collaborative, using the National Implementation Research Network’s five Active Implementation Frameworks (AIFs) (competency, organization, and leadership), as drivers of facilitation of rapid-cycle adaptations and implementation. The primary study outcome is hospital length of stay, with implementation metrics being used to evaluate adoption, fidelity, and sustainability. Additional clinical outcomes include opioid use, post-surgical complications, and post-discharge healthcare utilization (clinic/emergency room visits, telephone calls to clinic, and re-hospitalizations), as well as, assess patient- and parent-reported health-related quality of life outcomes. The protocol adheres to the Standard Protocol Items: Recommendations for Interventional Trials (SPIRIT) checklist.

**Discussion:**

The study provides a unique opportunity to accelerate the adoption of ERPs across 18 US pediatric surgical centers and to evaluate, for the first time, the effect of a pediatric-specific ENRICH-US intervention on clinical and implementation outcomes. The study design and methods can serve as a model for future pediatric surgical quality improvement implementation efforts.

**Trial registration:**

ClinicalTrials.gov NCT04060303. Registered on 07 August 2019.

## Background

Initiated in the 1990s, perioperative enhanced recovery protocols (ERPs) have progressively gained traction in a wide range of adult surgical disciplines and resulted in decreased hospital length of stay (LOS), in-hospital costs, complications, and markedly improved patient care experiences [1–7]. Yet, the implementation of ERPs in pediatric surgery is lagging and concerted efforts to demonstrate both clinical effectiveness and to examine obstacles to implementation are needed. Indeed, it is estimated that it takes nearly 20 years for evidence to make its way into clinical practice with failure rates for implementing complex innovations ranging from 30 to 90%, depending on the scope of organizational change involved [8–12]. Implementation can fail for many reasons, including misaligned incentives for adoption, unsustained leadership, lack of support and/or training, competing priorities, and resistance to change [13–16]. A supportive environment and implementation approaches that consider site-specific contextual adaptations and resources are necessary for effective implementation of interventions, such as ERPs [17].

This study will implement a modification for pediatric surgery of evidence-based, adult ERPs, the ENhanced Recovery In Children Undergoing Surgery (ENRICH-US) intervention. The study population will consist of pediatric patients undergoing non-emergent abdominal surgery, principally for inflammatory bowel disease (IBD), encompassing Crohn’s disease (CD) and ulcerative colitis (UC). These patients represent an ideal population in which to study the implementation of the ENRICH-US intervention because almost one-third of patients with CD present before age 20 and up to three-quarters of CD patients require gastrointestinal (GI) surgery for medically refractory disease [18, 19]. A quarter of UC patients present before age 20 and all patients with UC require colectomy to either manage severe disease or mitigate cancer risks [18, 20]. The GI surgical procedures performed in children with IBD are similar to the adult procedures for which ERPs have been well tested and validated.

Prior to initiating the study, a team of pediatric surgery experts adapted adult ERPs to meet the needs of pediatric patients undergoing elective GI surgery, primarily for IBD, by conducting (a) a systematic literature review, (b) a national survey to assess readiness for implementation, and (c) an expert panel adjudication of final pediatric ERP elements [21–23]. The study team also conducted a pilot study to assess the feasibility, safety, and preliminary effectiveness of the pediatric ERP in 79 pediatric patients who underwent elective GI surgery. Patient and provider education materials were created, a Learning Collaborative (LC) explored drivers and obstacles to implementation, a multidisciplinary team, including patient advocates, was engaged to enhance the quality improvement process, and data were routinely fed back to the implementation team members [24]. The pilot study demonstrated a ∼ 50% decrease in LOS, from 5 to 3 days, near elimination of intra-operative and post-operative opioid use, and a 30% decrease in perioperative fluid administration, without any increase in post-surgical complications or re-hospitalization [25].

### Elements of the ENRICH-US intervention

The basic elements of the ENRICH-US intervention are very similar to the elements of most adult ERPs and include perioperative counseling and education, maintenance of euvolemia through limited perioperative fasting and limited intra-operative fluid resuscitation, early enteral intake, early mobilization, limited opioid use, and non-routine use of surgical drains and tubes. Elements span the preadmission and pre-, intra-, and post-operative phases of care. Though each ERP element is independently simple, implementation of the combined elements likely will require substantial redesign of the systems and processes of care to assure a high level of coordination between surgery, anesthesia, and nursing clinicians.

**Fig. 1:**
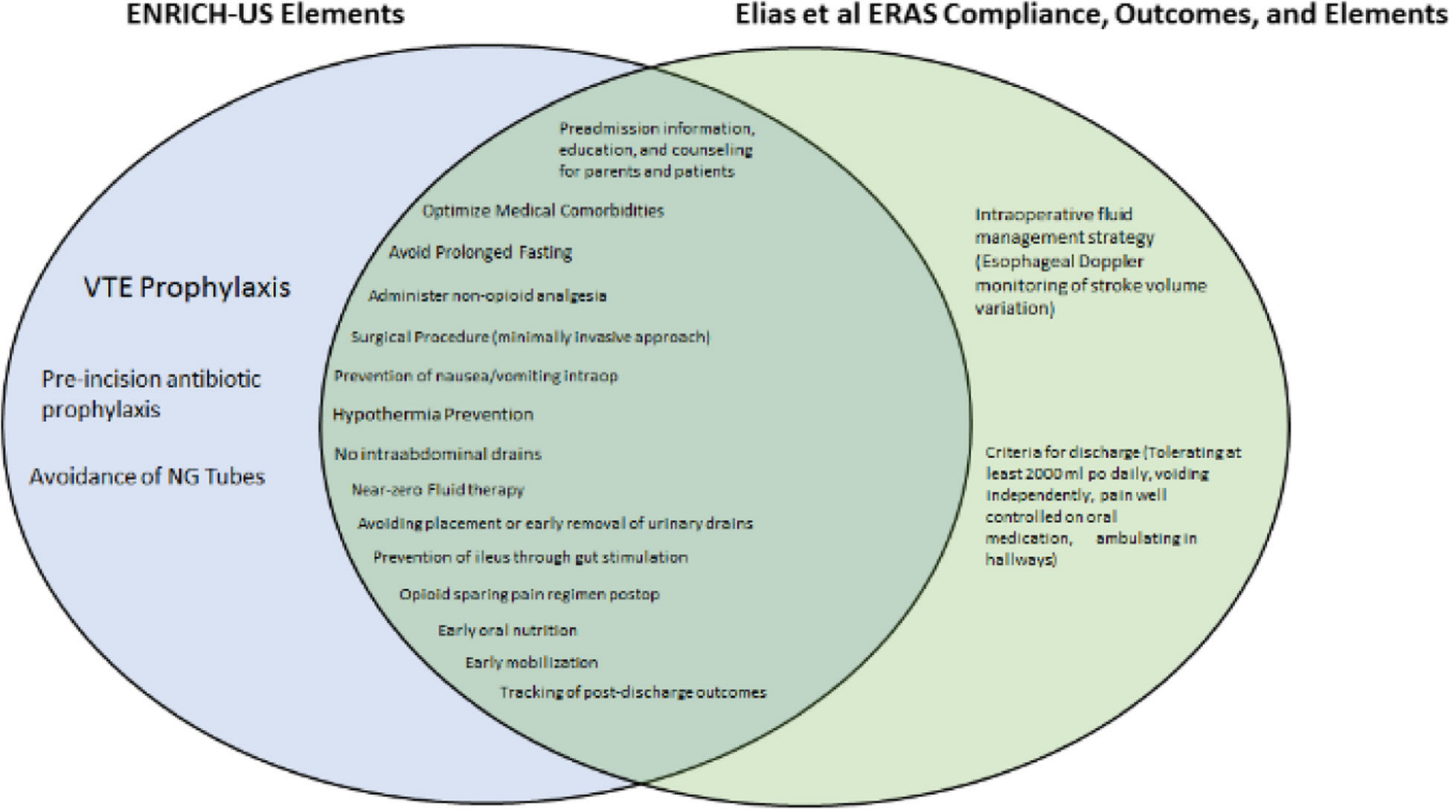
Comparison of elements in this study and elements suggested by ERAS and ERAS USA Society

The standard of excellence in reporting enhanced recovery after surgery is outlined by the Reporting on Enhanced Recovery Compliance, Outcomes, and Elements Research (RECOvER) Checklist (Fig. 1) [26], and Table 1 demonstrates the specific 21-elements included in the ENRICH-US intervention.

**Table 1:** ENRICH-US intervention elements

Pre-operative	Intra-operative	Post-operative
Patient and family education and engagement	Venous thromboembolism prophylaxis	No intraperitoneal/perianastomotic drains
Patient Advocate Liaison (PAL)	Pre-incision antibiotic prophylaxis	Goal-directed/near-zero fluid therapy
Provider education	Standardized anesthetic protocol	Avoiding or early removal of urinary drains
Optimize medical comorbidities	Surgical procedure (i.e., minimally invasive techniques)	Prevention of ileus through gut stimulation
Avoid prolonger fasting	Prevention of nausea/vomiting	Opioid sparing pain regimen
Administer non-opioid analgesia	Avoiding nasogastric tubes	Early oral nutrition
	Standardized hypothermia prevention	Early mobilization
		Audit protocol compliance/outcomes

## Methods/Design

### Study aims

The overall objective of the study is to demonstrate the impact of a pediatric surgery-specific ERP, entitled the ENRICH-US intervention. The specific aims of the study are to:

- Aim 1: Examine the effectiveness of the ENRICH-US intervention for elective GI surgery in pediatric patients:
  - Aim 1a: Measure the impact of the ENRICH-US intervention on clinical outcomes, including hospital LOS, opioid use, post-surgical complications, and post-discharge healthcare utilization (clinic/emergency department visits, telephone calls, re-hospitalizations);
  - Aim 1b: Assess patient- and parent-reported health-related quality of life (HRQoL) outcomes; and
- Aim 2: Assess implementation fidelity, sustainability, and site-specific adaptations of the ENRICH-US intervention for GI surgery by:
  - Aim 2a: Gather quantitative and qualitative measures of adoption, fidelity, and sustainability; and
  - Aim 2b: Identify organizational, leadership, and competency-based drivers of effective implementation and sustainability, using qualitative methodologies.

### Trial design and setting

**Fig. 2:**
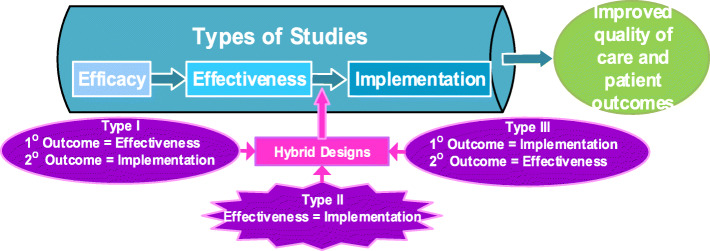
Hybrid study design with equal focus on clinical effectiveness and implementation

This prospective multiple site study uses a stepped-wedge, cluster-randomized, controlled study design to evaluate the implementation of the ENRICH-US intervention for pediatric patients who undergo elective GI surgery. A hybrid, type 2 study design will be used with equal focus on evaluating the effectiveness and the implementation (Fig. 2) [27–29].

**Fig. 3:**
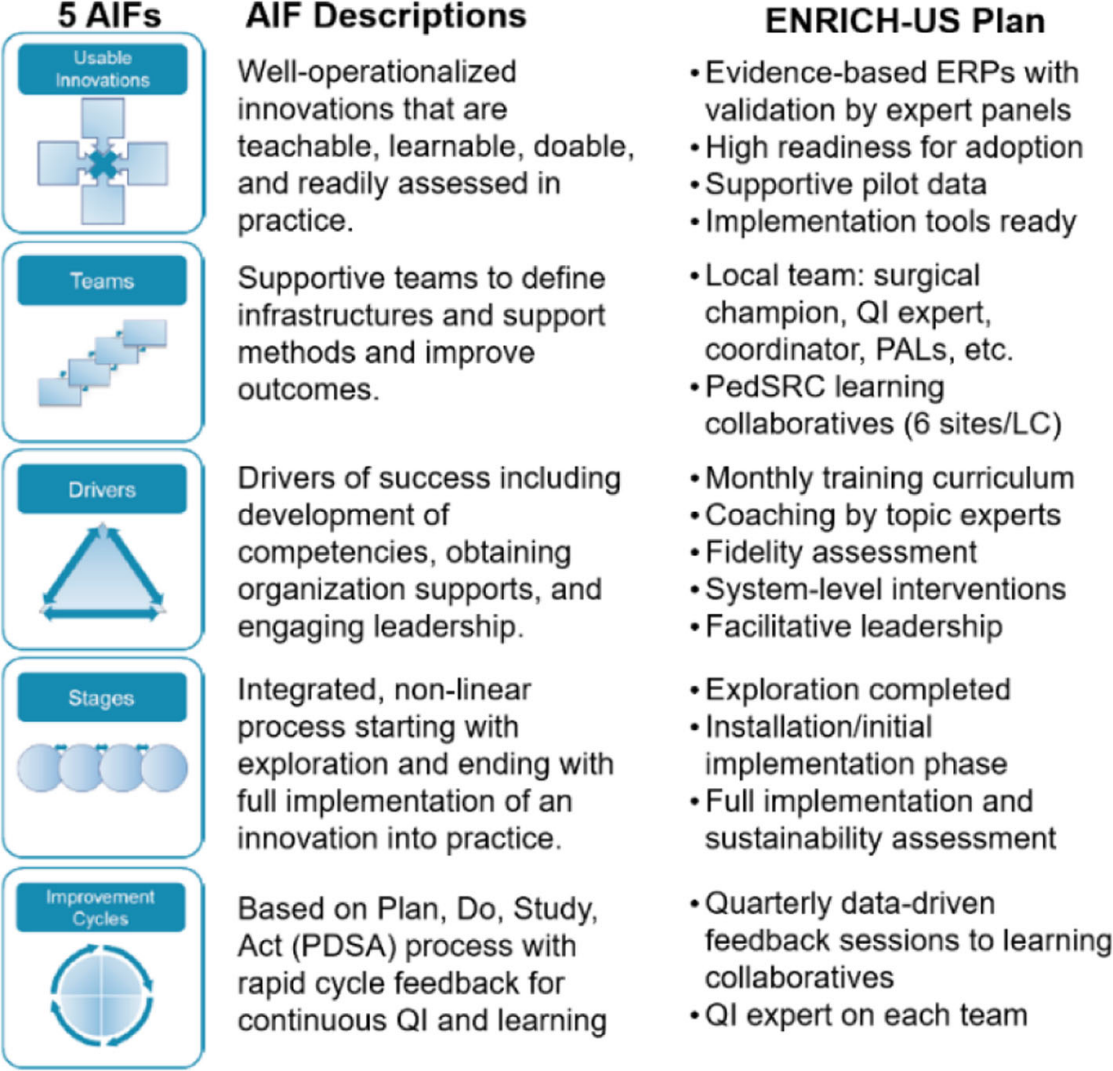
National Implementation Research Network’s five Active Implementation Frameworks AIFs to inform the implementation of ENRICH-US intervention

The study will facilitate contextual adaptation with rapid-cycle small tests of change using a LC approach that includes patient/parent stakeholders and sustainable and high fidelity implementation by applying the National Implementation Research Network’s five Active Implementation Frameworks AIFs (Fig. 3), which identify competency, organization, and leadership as drivers of implementation and empower team collaboration and evaluation [30]. The study is a pragmatic clinical trial, which will take place in the real clinical setting where patients receive usual care by their usual clinicians with data captured primarily from existing data sources, such as electronic health records (EHRs) and minimal study eligibility criteria for recruitment of pediatric patients undergoing non-emergent GI surgery [31]. The nature of this trial does not allow for subjects (patients or clinicians) to be blinded.

The trial is evaluating the effect of implementing a pediatric-specific ENRICH-US intervention for children recovering from surgery compared with usual care. All other perioperative care will not be modified from usual care pathways, including medications, and will continue for both trial arms.

The study will take place at 18 US hospitals (“sites”) that participate in the Pediatric Surgery Research Collaborative (PedSRC), a cooperative group of pediatric surgeons and researchers committed to performing clinical research in pediatric surgery (Table 2). All sites offer comprehensive, inpatient, pediatric services, including surgical services. The PedSRC represents one of the largest pediatric surgical networks for collaboration and research.

**Table 2:** Participating children’s hospitals

1.	Seattle Children’s Hospital, University of Washington
2.	Doernbecher Children’s Hospital, Oregon Health and Science University
3.	Children’s Hospital of Los Angeles, University of Southern California
4.	Primary Children’s Hospital, University of Utah
5.	Dallas Children’s Hospital, University of Texas Southwestern Medical Center
6.	Children’s Memorial Hermann Hospital, University of Texas at Houston
7.	Texas Children’s Hospital, Baylor College of Medicine
8.	LeBonheur Children’s Hospital, University of Tennessee at Memphis
9.	Ann and Robert H. Lurie Children’s Hospital of Chicago, Northwestern University
10.	Riley Children’s Hospital, Indiana University/Purdue University
11.	Shands Children’s Hospital, University of Florida
12.	MUSC Children’s Hospital, Medical University of South Carolina
13.	Duke University Children’s Hospital and Health Center, Duke University
14.	Children’s Hospital of Richmond at VCU, Virginia Commonwealth University
15.	John R. Oishei Children’s Hospital, State University of New York at Buffalo
16.	Cohen Children’s Medical Center, Feinstein Institute for Medical Research
17.	Alfred I. duPont Hospital for Children
18.	Children’s Hospital Boston, Harvard University

### Study populations and inclusion and exclusion criteria

The study involves two study populations. One consists of pediatric patients, ages 10–18, who need to undergo a non-emergent GI procedure, and their legal guardian(s) or parent(s). The other consists of all clinicians who are involved in the care of pediatric patients undergoing GI surgery and who will be part of the LC. Children requiring emergent GI surgery and patients/families who cannot read and write English or Spanish, as all materials/surveys will be available in English and Spanish only, will be excluded. Inclusion or exclusion of subjects will not differ based on sex/gender or race/ethnicity.

#### Pediatric assent and adult/parental/legal guardian consent

Authorized study team members at each site will be responsible for screening, recruiting, and consenting eligible subjects. The consent process will be executed for all subjects before any study data are collected. Once a subject is deemed eligible and has signed the assent (if the subject is ≥ 18 years old he/she will sign the consent), and their parent/legal guardian has signed the informed consent and permission form (ICF), the subjects are considered to be enrolled in the study. The pediatric assent and adult consent forms will be IRB approved, either on a printed form or electronically through REDCap (eConsent), and may occur in-person or through Telehealth. On the consent form, subjects will be asked to agree to the use of their data should they choose to withdraw from the trial. Subjects will also be asked for permission for the research team to share with any required entities from the institutions taking part in the research or regulatory authorities, when required. This trial does not involve collecting any biological specimens for storage.

### Randomization and timeline

**Fig. 4:**
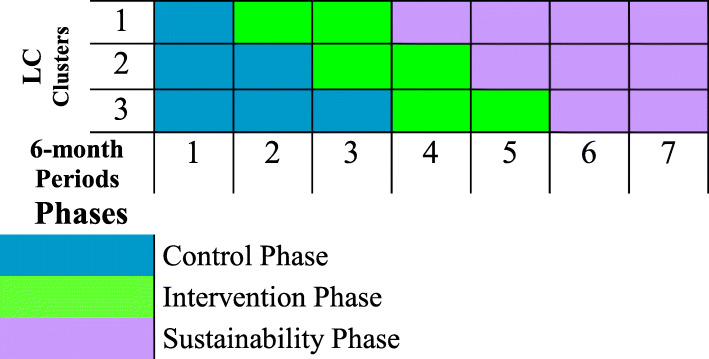
Stepped-wedge cluster trial design

**Fig. 5:**
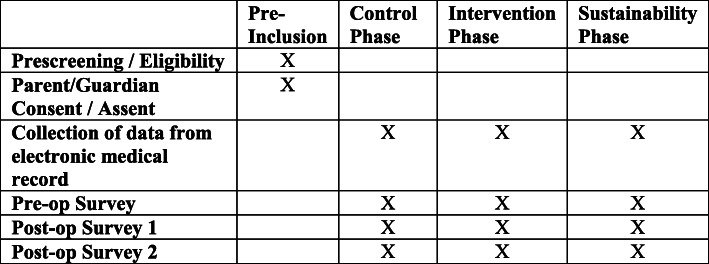
Time schedule of enrollment for participants

The 18 participating sites will be randomly assigned by the study statistician to one of three clusters for the stepped-wedge design, with each cluster consisting of six sites, in turn, being randomly assigned to an intervention start period [32–34]. Given that many sites have already initiated some ERP elements, a study design that randomizes sites or patients to a control arm without any ERP elements is no longer feasible [33, 34]. Figure 4 shows the study design. All sites will initially have at least a 6-month “control” phase during which baseline, pre-intervention data will be collected. Then, each cluster will have a 12-month ENRICH-US implementation or “intervention” phase. Post-intervention data will be collected during a “sustainability” phase of at least 1 year and, for up to 2 years at some sites. Each cluster will be a LC to collectively share and learn from each other about contextual adaptations and implementation strategies, guided by the five AIFs. Figure 5 shows the time schedule of enrollment for subjects.

To improve the generalizability of the study’s findings, the study purposefully included diverse sites, including some low-volume pediatric surgical sites. Exploring implementation obstacles unique to low volume sites will facilitate future dissemination efforts beyond the participating sites. To reduce the potential bias of surgery volume among the sites, a stratification randomization schema will be used [35]. Based on our initial assessment of surgical volumes, there are 12 high-volume sites (range 20–120 cases/year) and 6 low-volume sites (< 20 cases per year). Thus, we propose to employ a stratification randomization schema such that each of the 3 clusters has 4 high-volume sites and is limited to 2 low-volume sites.

### Study phases

#### Installation phase

Training for the “control phase” data collection of the site PI, research coordinator, and data abstractors will take place during the first two quarters of year 1. Training will focus on the creation of site-specific workflows for patient recruitment, enrollment criteria, consent processes, and the use of data abstraction tools. Given the stepped-wedge design, each site PI and research coordinator will also recruit and create a site implementation team, 6–9 months prior to the cluster’s intervention start period, to optimize team engagement. For example, each cluster will establish their teams in 6-month intervals (e.g., cluster 1 in the second quarter of year 1; cluster 2 in the final quarter of year 1; and cluster 3 in the second quarter of year 2). Each site implementation team will include, in addition to the site PI and study coordinator, at least one anesthesia champion, one patient advocate liaison, and one hospital-level QI leader.

Similarly, a monthly video-conference LC meeting with each cluster will begin about 1 month after each site in the cluster has created their implementation team. The LC approach includes sharing of obstacles and barriers to implementation of specific elements, reporting on the use of implementation toolkit contents (e.g., order sets, patient education materials) to assist with implementation, providing updates about rapid cycle small tests of change with data feedback, and learning about and sharing strategies to overcome barriers to implementation. All LC video-conference sessions will be audio-video recorded and serve as a source of data that will be analyzed qualitatively to provide a catalog of the multiple contextual adaptations and strategies undertaken by sites to optimize adaptation and implementation.

#### Control phase

This phase encompasses the AIF installation phase. All clusters begin the control phase at the same time but continue for varying periods (6–18 months). Each site coordinator will recruit, screen, enroll, and consent eligible patients. This phase consists primarily of individual-level patient data collection. Patients and parents will complete web-based HRQoL assessments preoperatively, at 2–5 days, and at 4–6 weeks, post-operatively. This phase involves abstraction of existing EHR data by the site coordinator about current, standard perioperative care received by patients. Site-specific barriers and facilitators to implementation will be assessed by semi-structured, telephone interviews, conducted with each site’s PI and coordinator.

During the control phase, standard perioperative surgical care will be provided. Some elements of the ENRICH-US intervention may already be standard of care and, during the control phase, these elements will continue to be offered and details about the use of these elements will be collected.

#### Implementation phase

Each cluster will be randomized to an intervention phase start date. Toolkits for patients (e.g., patient education materials) and for providers (e.g., standardized order sets), developed during the feasibility pilot study, will be shared with the Cluster LCs to facilitate the adoption of the tools. During the monthly, video-conference Cluster LC meeting, (a) a didactic session, consisting of a curriculum, derived from prior research will be delivered; (b) a discussion by the six sites of obstacles and barriers, as well as, of effective strategies for implementation; and (c) a reporting and sharing of preset measures of implementation.

Risks/benefits related to the patient’s surgery will be reviewed by the surgeon and anesthesia/pain teams prior to surgery. Clinical and ERP intervention-specific complications/side effects will be closely monitored by each site’s clinical care teams and deviation or discontinuing from ERPs for clinical necessity will be at the discretion of the patient’s care providers and surgeon.

#### Sustainability phase

The stepped-wedge approach enables documentation and evaluation of sustainability for up to 2 years at some sites, offering a unique opportunity to assess the intervention’s effect well beyond initial implementation. This phase also allows for assessment of both the impact of the intervention and fidelity of implementation and sustainability.

### Outcome measures

Outcome measures are summarized in Table 3. For Aim 1, the primary outcome of clinical effectiveness is hospital LOS, with secondary outcomes of opioid use, post-surgical complications, and post-discharge healthcare utilization (clinic/emergency department visits, telephone calls to clinic, and re-hospitalizations) [36–42]. The primary outcomes of Aim 2 consist of implementation metrics, including adoption, fidelity, and sustainability of the ENRICH-US intervention [43]. Secondary outcomes consist of the contextual factors associated with ENRICH-US intervention implementation, captured during the LC sessions.

**Table 3:** Effectiveness and implementation outcomes

	Aim 1 - clinical effectiveness	Aim 2 - implementation
**Test**	ENRICH-US Intervention	Implementation strategy
**Analysis**	Patient- and hospital-level assessment	Surgeon- and hospital-level assessments
**Theoretical outcomes**	Health outcomes; process/quality measures are considered intermediate steps	Adoption/uptake of the clinical intervention; process/quality measures
**Primary outcomes**	Length of stay (LOS) for Aim1a	Implementation metrics including adoption, fidelity, and sustainability for Aim2a
**Secondary outcomes**	Decreased intra-operative fluid use^a^, decreased intra-operative and post-operative opioid use^a^, shorter time to regular diet^a^, health care utilization, surgical complications^b^, HRQoL assessment for Aim 2b.	Contextual factors associated with implementation including modifications and adaptations to materials, implementation barriers, implementation outcomes, and feedback on challenging ERP elements at site level for Aim2b

^a^These outcomes are specifically addressed by ERP elements and interventions

^b^Complications are defined as per ACS NSQIP Pediatric definitions

### Data collection

For Aim 1, the Northwestern University (NU) Data Coordinating Center (DCC) will work closely with each site’s PI and coordinator to set-up access to data collection tools in the Research Electronic Data Capture (REDCap) system. The DCC will provide web-based training tutorials. Only the site PI, coordinator, and data abstractors will be able to identify patients at their site. Extensive training will prepare each site’s coordinator/data abstractor to monitor enrollment, enrollment targets, and compliance with eligibility criteria; monitor completion of data collection and send additional reminders; and produce performance reports for the monthly LC video-conference meeting (e.g., enrollment per month, compliance with the ENRICH-US intervention elements). Coordinators/data abstractors will be provided with a data dictionary with definitions of all study measures.

Measures include patient demographic (e.g., age, sex, race/ethnicity) and surgery-specific information (e.g., surgeon, diagnosis). At the start of the study data collection, the first five cases at each site will be used to assess data quality and any additional training needs, in close collaboration with the DCC. Coordinators/data abstractors will also receive training on the collection of HRQoL assessments (pre-, 2–5 days post-, and 4–6 weeks post-operatively). Sites will provide support for patients/parents who will be asked to complete the REDCap-based surveys by telephone, iPad, or computer with paper versions available, as needed.

For Aim 2, implementation metrics and contextual factors needing adaptations will be collected by recording the monthly LC video-conference meetings. Qualitative methods will be used to categorize key adaptation themes and implementation strategies. Additional semi-structured interviews will be conducted by the research team with each site PI and coordinator.

### Analytical approaches

#### Sample size

The study is powered to detect a significant difference in hospital LOS between patients who receive the ENRICH-US intervention (cases) and patients who receive standard post-operative care (controls). Calculation of the anticipated power was based on the design effect of a stepped wedge, clustered randomized controlled study [44]. The design effect is based on the number of clusters, the intra-cluster correlation coefficient, and the average cluster size per period. A total of 18 hospitals participating in the PedSRC agreed to participate in the study. Prior data indicates that approximately 616 GI surgeries are performed annually across the sites, for an average of 34 surgeries per site (range 5–120 patients per site per year). Overall, a sample of an average of 20 patients per site provides > 80% power to detect a difference in LOS of at least 1.25 days, and a 60% enrollment rate will yield an adequate sample for this study.

#### Statistical analysis of Aim 1

Descriptive statistics will be calculated for all variables of interest and include means with standard deviations, medians and ranges, or counts and percentages, as appropriate. Characteristics of the clusters and of patients within each cluster will be summarized by group of stratification randomization. Effect sizes will be used to quantify the degree of clinical effectiveness for the primary outcome (LOS) and secondary outcomes.

The primary analytic approach is the use of generalized linear mixed models (GLMM). These models allow for the estimation of both fixed effects (e.g., intervention, cluster) and random effects which include the variation of individual clusters around the conditional mean of the clusters [45]. Models will include a hospital-specific random intercept, using an unstructured covariance matrix, if possible. Two-level (patients/hospital) and three-level (patients/physicians/hospitals) models will be considered in the analysis, when possible. Appropriate modeling assumptions will be verified, and data may be transformed (e.g., logarithm, square-root) to meet model assumptions. All effect sizes (e.g., difference in means, rates) will be presented with associated 95% confidence intervals. Because calendar time may be associated with both exposure to the intervention and, possibly the outcome, it will be included as a potential confounder in all analyses. In addition, the length of the period during which the cluster has been exposed to the intervention will be analyzed as a possible effect modifier [46].

In our primary analysis, patients will be classified into treatment groups, based on each site’s randomized crossover time and analyzed, using the intention to treat principle. In a sensitivity analysis, we will examine the effect of the ENRICH-US intervention “dose” by using the number of elements delivered to a patient, as a treatment adherence measure. Heterogeneity of treatment effects (HTE) will be examined, using a within-cluster comparison of exposed and unexposed periods which will help to identify clusters and cluster characteristics that may inform aspects of the adaptation and sustainability. The analysis will be conducted using SAS v. 9.4 (Cary, NC) and R v.3.2.2 (Vienna, Austria), and the statistical significance level will be considered 0.05 unless otherwise noted.

#### Qualitative analysis of Aim 2

The LC meeting recordings and semi-structured interview transcripts will be coded and analyzed thematically, using a hybrid form of textual analysis which combines inductive and deductive logics, and informed by the tasks at hand (implementation of ENRICH-US intervention, degree of implementation fidelity, site-specific, contextual adaptations), as well as, allowing for unanticipated themes and subject perspectives to emerge, as in our prior work [47–50]. A computer-assisted qualitative data analysis software (MAXQDA) will be used with initial categorical themes being entered and applied to further transcripts. Emerging themes will be iteratively checked with the interviewees to ensure the accuracy, completeness, and perceived validity.

The DCC will use qualitative comparative analysis (QCA) to combine the qualitative and quantitative data and determine the necessary and sufficient conditions for successful implementation [51, 52]. QCA answers the question: What conditions, alone or in combination with other conditions, are necessary or sufficient to produce the outcome of interest? QCA is a case-oriented, mixed-methods approach of analysis that examines relationships between conditions and an outcome of interest, which captures the complexity of implementing QI interventions. Accordingly, QCA allows for systematic, cross-case comparison of data for implementation purposes [53]. For the QCA analysis, quantitative data, such as hospital setting (e.g., rural), bed size, number of surgeons, and case volumes, will be merged with the qualitative results. Using QCA, we will be able to identify the causal pathways to the outcome(s) of interest and identify conjunctional causation or the conditions that may only display their effects in conjunction with other conditions [54].

### Trial management

#### Roles and responsibilities

The study sponsor/management and DCC is located at Northwestern University (NU). The DCC research team will perform study management tasks such as site management and contracting, central Institutional Review Board (cIRB) submissions and oversight, creation of the data entry database, trial-wide communication, orchestration of site PI and coordinator training activities, coordination of site initiation, creation and delivery of LC meeting webinars about the ENRICH-US intervention, leading the monthly LC meetings during the implementation phases for each cluster, and performing all data analyses. Monitoring will be performed by the DCC centrally. The DCC uses web-based data validation rules, data manager assurance of data collection through independent double entry, routine statistical analysis, and on-going review of site metrics. Remote monitoring will include verification of written consent and study enrollment, by monthly and quarterly periods. To verify informed consent forms of enrolled subjects, sites will upload an electronic portable document format (PDF) of the signed consent form into the password-protected REDCap database. The PDF file will be linked to the subject identification and stored on a secure server behind the NU firewall. The files on these servers can only be accessed by designated study personnel upon entry of a password. The DCC will remotely monitor the informed consent forms and any identified issues will be relayed to the site for corrective and preventive action. Significant protocol amendments will be addressed with the study’s funding agency first, and then submitted to the cIRB for approval. Minor protocol amendments will also be submitted to the cIRB for approval and distributed to the sites, thereafter. Additionally, the protocol will be updated in the clinical trial registry.

#### Data quality assurance and quality control

Routine data audits will be performed to monitor data completion and integrity by the DCC. The DCC will verify data for a random sample of 10% of enrolled patients at each site. Data quality assurance processes at the DCC include logic and rule checks built into the REDCap database and real-time central monitoring. Quality control (QC) procedures will be implemented beginning with the data entry system, and data QC checks that will be run on the database will be generated. Any missing data or data anomalies will be communicated to the site(s) for clarification/resolution.

#### Ethical considerations and data security

All investigators will ensure that the study is conducted in full conformity with Regulations for the Protection of Human Subjects of Research codified in 45 CFR Part 46, 21 CFR Part 50, and/or 21 CFR Part 56.

Pediatric GI surgeries have complication rates of 20 to 40% [55, 56]. While each of the ENRICH-US intervention elements has some inherent risk, patients were purposefully selected as study subjects because they most closely resemble adult patients for whom the safety of ERPs has been well established. In addition, all participating surgeons retain the right to deviate from the ENRICH-US intervention for any clinical need. While consent/assent will be obtained from patients and their parent/legal guardian, they will retain the right to withdraw from the study at any time without any effect on their healthcare.

To avoid the identification of individual patients, all data feedback to sites will be performed in an aggregate fashion. While site level-performance measures will be used during the LC meetings and iterative processes of improving implementation, patient-level and provider/surgeon-level data will only be provided and/or discussed with each site. Similarly, any peer-review publications or subsequent dissemination of results will only include aggregate results reported by site, with all sites remaining anonymous.

Data Security is of the upmost importance and the DCC, under the purview of the two Co-PIs, will assume responsibility for the Data Security Plan. All data collected in the REDCap system will be initially stored securely in the NU enterprise data warehouse (EDW), which is HIPAA-compliant. Subsequent data downloads from the EDW for use by the DCC will be stored on research servers that have restricted access and require institutional login and passwords. Similarly, all audio recordings and transcriptions of audio from interviews and WebEx sessions will be saved in secure research servers at NU.

Two Co-PIs are responsible for granting data access only to DCC staff critical to the conduct of the study. For data backup and recovery, all source study data are stored on secure serves at NU and within the NU EDW. These systems have backup and recovery systems in place in case of equipment malfunction, physical facilities impairment, or natural disaster.

#### Data retention and archiving

Once the research project is completed, all study data will be stored and secured for the length of time required by the award (minimum 3 years) with additional extension as deemed needed by the study Co-PIs, based on ongoing secondary analyses of the data. At this juncture, data will be removed from the NU EDW and secured with password-protected and user-access restricted research data drives/servers at NU. As per National Institutes of Health requirements, a research dataset will be created that is stripped of all patient health information and personally identifiable information for more public, generalized use.

#### Oversight and monitoring

A Data Safety and Monitoring Board (DSMB) will ensure the safety of subjects and the validity and credibility of the trial. This board will include an experienced pediatric gastroenterologist, a pediatric surgeon, and a patient or parent of a patient who underwent GI surgery. All board members are not involved in the study and are free of competing interests. Interim analyses will be conducted every 7 months and include any potential adverse events (event rates), timing and completeness of data collection, adequacy of sample size, comparability of treatment clusters [35]. The DSMB can ask for more frequent reporting if concerns arise. Approximately 2 weeks before the DSMB meeting, the study statistician will send a report to the DSMB members that includes patient recruitment, ratio of consent to randomization, attrition, and any adverse events by treatment, labeled A or B. While the study is not blinded by design, the data will be presented in a blinded fashion to reduce any bias associated with knowing the intervention cohort. The board will review the results and assure that the conduct of the trial is acceptable. Any recommendations of the committee will be directly made to the Co-Principle Investigators. The trial will be stopped if the committee identifies any significant safety concern which warrants stopping the trial.

## Discussion

This study protocol will allow for the evaluation of the effectiveness, implementation, and sustainability of all the recommended elements of the ENRICH-US intervention. Prior pediatric surgery studies have typically involved ≤ 6 ERP elements, while most adult ERPs have more than 20 elements. The study has been designed and powered to detect a decrease in LOS between cases (receipt of ENRICH-US intervention) and controls (receipt of standard post-operative care). Improvements in a wide variety of secondary outcomes, such as opioids use, post-operative complications, and patient/family HRQoL may also occur. Regardless of the effectiveness, this study will reveal important information about the uptake and sustainability of a complex intervention at a wide range of pediatric surgical centers. This information will be highly informative for future, large-scale QI efforts in pediatric surgery. Tangible outputs include the creation of implementation toolkits and coaching videos. These efforts align with the shift in national health policy from a focus on quantity to a focus on quality and value-based care. The patient-reported outcomes provide useful insight to the specific aspects of care most highly valued and prioritized by patients and families. In addition, prior pediatric studies have had limited acknowledgement of bias, inconsistent outcomes, and lacked adequate controls.

While the success of this study has far-reaching implications for advancing pediatric surgical care and improving outcomes for pediatric patients undergoing GI surgery, next steps include expansion and examination of a similar ERP for younger patients, for broader populations including critical care and care of medically complex pediatric populations, and for a wider-spectrum of pediatric surgical procedures.

### Trial status

This study can be found on ClinicalTrials.gov under the registration number NCT04060303. The current protocol Version 2.0 20-Jul-2020. Enrollment began July 2020, and recruitment will continue for 3.5 years.

## Data Availability

The study team plans to write up study results for publication in scientific journal articles to reach healthcare professionals. Dissemination of the results of the study is an essential part of the proposed activities and the dissemination plan includes multiple approaches. The investigators wish to make the results available both to the community of clinicians who care for IBD GI patients and to the patients and families with IBD. A key goal of the dissemination plan will be to prepare appropriate materials, as soon as data are valid and reliable. Study results will also be posted on ClincialTrials.gov. All data collected during the study will be made available in a de-identified manner. In addition to the peer-reviewed publication of study results and the dissemination of findings through publicly available websites, blogs, and newsletters, the PI and Biostatistics Director will create a patient de-identified data set that will be made publicly available to researchers. These public files will include clinical data and outcomes collected through REDCap for the complete study, as well as raw data for the HRQoL surveys. All data will be fully de-identified at the patient- and hospital-level. Data requests will be made to the PI (via direct email contact or through the study website) who will arrange for secure transfer of data through a secure web-based data sharing tool (such as Dropbox). A copy of the study protocol, data definition dictionary, and public use file with instructions will also be generated for ease of use. Data will be made available in a format that will easily be used in mainstream software analysis packages (e.g., ASCII, SAS). The data will be made available as soon as possible but no later than 1 year of the completion of the funded study period for the parent award or upon acceptance of the data for publication, whichever is earlier. All individually identifiable health information will be stripped from the public use data sets, as per Health Insurance Portability and Accountability Act (HIPAA) guidelines, as well as, the omission of indirect identifiers that are not listed as patient health information but could lead to “deductive disclosure” such as comment fields and study site numbers. Furthermore, participating sites will be protected by stripping all actual names and creating a random number to represent data collected from each site.

## Abbreviations

ACS NSQIP: American College of Surgeons National Surgical Quality Improvement Program
AIFs: Active Implementation Frameworks
APSA: American Pediatric Surgical Association
CD: Crohn’s disease
CFR: Code of Federal Regulations
DCC: Data Coordinating Center
EDW: Enterprise Data Warehouse
EHRs: Electronic health records
ENRICH-US: ENhanced Recovery In CHildren Undergoing Surgery
ERAS: Enhanced recovery after surgery
ERPs: Enhanced recovery protocols
GI: Gastrointestinal
IBD: Inflammatory bowel disease
HRQoL: Health-related quality of life
LC: Learning Collaborative
LOS: Length of stay
NGT: Nasogastric tube
NU: Northwestern University
PDF: Portable Document Format
RECOvER: Enhanced Recovery Compliance, Outcomes, and Elements Research
REDCap: Research Electronic Data Capture
PedSRC: Pediatric Surgery Research Collaborative
QC: Quality control
QCA: Qualitative comparative analysis
UC: Ulcerative colitis
VTE: Venous thromboembolis
